# A new species of *Patania* from the Hainan Island, China (Lepidoptera, Crambidae)

**DOI:** 10.3897/zookeys.614.8810

**Published:** 2016-09-01

**Authors:** Dan Xu, Xi-Cui Du

**Affiliations:** 1 College of Plant Protection, Southwest University, Chongqing, China

**Keywords:** Diaoluo Mountain, Pyraloidea, Spilomelinae, taxonomy

## Abstract

*Patania clava* **sp. n.** is described from the Diaoluo Mountain of Hainan Island, China. The new species is distinguished from its most similar congener, *Patania iopasalis* (Walker, 1859), by the following features: wingspan 33.0–35.0 mm (vs. 21.0–30.0 mm), ventral cilia of the male antenna as long as the diameter of flagellomere (vs. 1/4), the thick finger-like gnathos (vs. the short broad sheet-like), a long thick needle-like cornutus stretching out from (vs. embedded in) a cluster of spicular cornuti near apex. Images of adult and genitalia of the new species are provided.

## Introduction

The genus *Patania* was established by Moore (1888) based on the type species, *Botys concatenalis* Walker, 1866, from Darjeeling, India. Warren described another new *Patania* species in 1896, *Patania verecunda* (Warren, 1896). Meanwhile, Hampson (1896) regarded *Patania* and *Pleuroptya* Meyrick, 1890 as synonyms of *Sylepta* Hübner, 1823. Hampson’s opinion was followed by most researchers (Hampson 1898; Shibuya 1928, 1929; Klima 1939; Bae et al. 2008) except for Rose and Singh (1989) who published a new species of *Patania* separately. Inoue (1980) revalidated *Pleuroptya*, and some new species, sub-species, and combinations of *Pleuroptya* were recorded since (Yamanaka 1995, 1998; Munroe 1983; Shaffer and Munroe 1989; Leraut 2005). Kirti and Gill (2007) considered *Patania* and *Pleuroptya* congeneric by comparing the morphological characters and male genitalia of their respective type species. The generic name *Pleuroptya* Meyrick, 1890 was suppressed as subjective synonym of *Patania* Moore, 1888 by Kirti and Gill (2007).

Globally, there are approximately 50 described species of *Patania* (Kirti and Gill 2007; Li et al. 2009, Nuss et al. 2003–2015), 28 species being recorded in China (Li et al. 2009; Xu 2015). In this paper, *Patania clava* sp. n., collected from the Diaoluo Mountain, Hainan Island, China, is described as new to science.

## Materials and methods

Specimens were collected by 250-W high-pressure mercury lamps. They were hand-collected alive and killed by ethyl acetate. The type specimens of *Patania* species deposited in the Natural History Museum of London (NHM) have been examined by corresponding author. All the type specimens of the new species are deposited in the College of Plant Protection, Southwest University, Chongqing, China (SWUCPP).

The terminology mainly follows Kristensen (2003) and Slamka (2013).

Genitalia preparation followed the procedure of Li and Zheng (1996), using boiling 10% NaOH solutions to digest internal tissues; after careful cleaning and removal of scales and content of coelom, genitalia were examined, compared, and described before being mounted on microscope slides by the first author. The images of the adults were taken with a digital camera (Nikon P7700). The illustrations of the genitalia were prepared with a digital camera Leica DFC 450 attached to a digital microscope Leica M205 A.

## Taxonomic account

### Patania clava sp. n.

Taxon classificationAnimaliaLepidopteraCrambidae

http://zoobank.org/3DEA6F10-06C7-4ABB-BE34-97C03A4E4729

Figs 1–4 , 9–10

#### Holotype.

male, China, Hainan, Mt. Diaoluo, 109.87°E, 18.72°N, 900 m, 23.V.2014, leg. Li-Jun Xu & Dan Xu, pinned, slide number XD15056. Original label: “Hainan, Diaoluo Mountain, vocational village, 900 m, 23.V.2014, Li-jun Xu & Dan Xu”.

#### Paratype.

1 female, same data as holotype, slide number XD15050.

#### Diagnosis.

**Figures 1–4.**
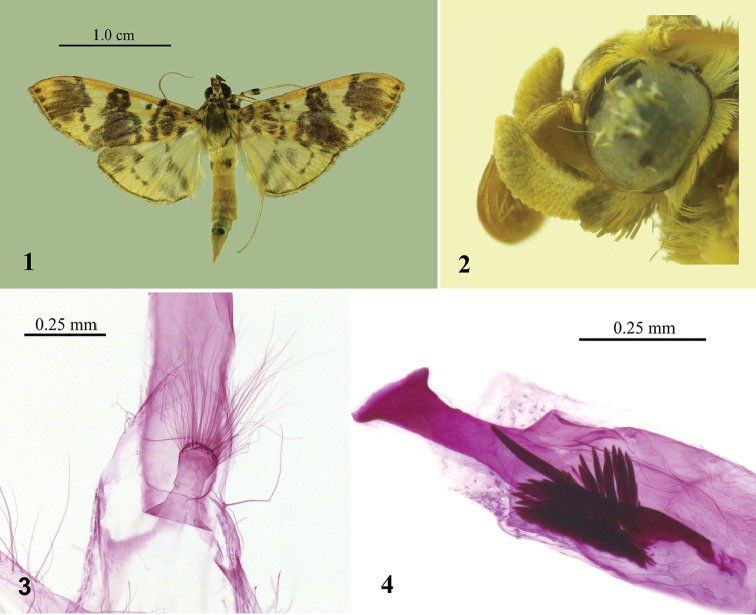
*Patania clava* sp. n., Holotype. **1** Adult **2** Head **3** Gnathos **4** Cornuti.

This species can be distinguished by the wingspan of 33.0–35.0 mm (Fig. 1); the brown patch near base of the labial palpus (Fig. 2), the male antenna with ventral cilia nearly as long as the diameter of flagellomere; in the male genitalia by the thick finger-like gnathos bearing long setae on the apex (Fig. 3), the phallus with a dorsally protruding sclerotized structure with a slant nailhead-like apex, and a long thick needle-like cornutus stretching out from a cluster of spicular cornuti near apex (Fig. 4).

#### Description.

Adult (Figs 1–2). Wingspan 33.0–35.0 mm. Body pale yellow. Antenna pale yellow dorsally, orange ventrally. Male antenna with ventral golden cilia nearly as long as the diameter of flagellomere. Labial palpus upturned, pale yellow with a brown patch near base. Maxillary palpus small, pale yellow. Patagium pale yellow, with large fuscous spots. Tegula pale yellow and fuscous. Forewing pale yellow, with fuscous lines, spots and patches; a spot at inner edge near basal line; a large elliptical patch between basal line and antemedial line; orbicular stigma distinct; discoidal stigma reniform, its posterior part overlapping postmedial line partly; a large patch outspread between discoidal stigma and inner margin; postmedial line oblique inward from costa, excurved vertically from M_1_ to CuA_2_, with a distinct punctiform pattern between M_2_ and CuA_2,_ then sharply incurved along CuA_2_ as an indistinct line before curving towards the dorsum; a large patch between anterior half of postmedial line and marginal line, marginal line consisting of a line of spots. Hindwing pale yellow; discoidal stigma brown; a brown slant stripe below end of discal cell and a pale brown fuzzy band beyond it; postmedial line oblique inward from costa, excurved vertically from M_1_ to CuA_2_, with a distinct punctiform pattern between M_2_ and CuA_2_, then sharply incurved along CuA_2_ as an indistinct line before curving towards the dorsum; marginal line fuscous; brown blot near apical angle. Fringes of forewing and hindwing white at base, pale brown terminally. Fore coxa yellow, with two big black spots; femur yellow except for black distal end; tibia pale yellow with distal half black, tibial comb orange. Mid femur white, with a black spot near centre; tibia pale yellow, black distally, a black spot near the base. Hind femur white, with a brown spot near the distal end; tibia white, somewhat pale brown near the base. Abdomen orange dorsally; anterior edge of 2^nd^ segment with two lateral black spots flanking the centre, 7^th^ segment with a big black spot separated slightly at the center; pale yellow ventrally.

**Male genitalia.** (Figs 3–4, 9). Uncus triangular, blunt on posterior margin. Gnathos thick finger-like, apex circularly widened and dorsally studded with long thin simple setae. Valva ligulate. Fibula near base of valva, flat, triangular, with long dense setae. Transtilla triangular, connected medially, with sparse setae. Saccus oblong, with the anterior apex rounded. Juxta ovate. Phallus cylindrical, with a dorsally protruding sclerotized structure with a slant nailhead-like apex, a long thick needle-like cornutus stretching out from a cluster of spicular cornuti.

**Female genitalia.** (Fig. 10). Apophysis anterioris about 1.5 times length of apophysis posterioris, triangular extension near base unilaterally. Ductus bursae about 4 times length of corpus bursae, middle ductus bursae widened, area posterior of widening slightly sclerotized; ductus seminalis originating at posterior end of ductus bursae. Corpus bursae elliptical, without signum. Both ductus bursae and corpus bursae densely studded with tiny granules.

#### Etymology.

The specific name is derived from the Latin *clavus* (nail), in reference to the nail head-like apex of sclerotized structure of the phallus.

#### Distribution.

This species is only known from the Diaoluo Mountain of Hainan Island, China.

#### Natural history.

Unknown except that the moths fly late May and are attracted to light. The habitat in which this species has been collected is located at an altitude of 900 m. The vegetation of the habitat is a blend of shrubs, conifer trees and broad leaved trees.

#### Remarks.

**Figures 5–8.**
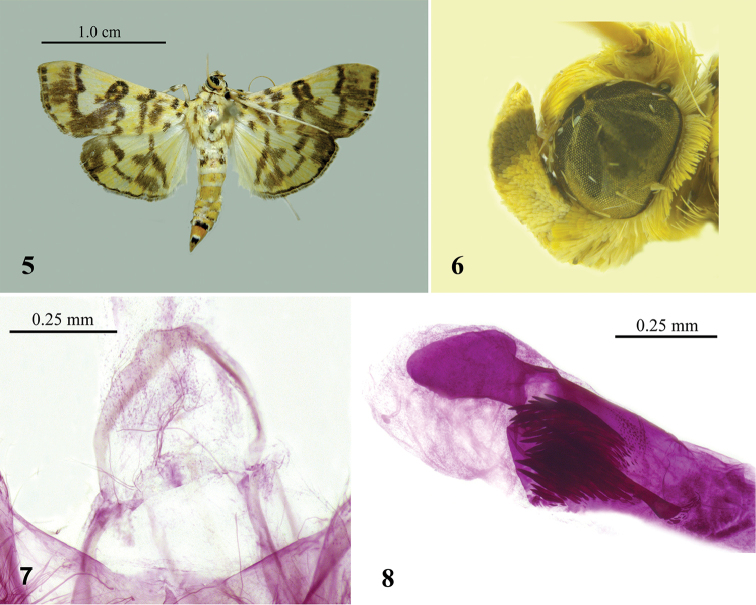
*Patania iopasalis* (Walker). **5** Adult **6** Head **7** Gnathos **8** Cornuti. Slide number XLJ14083.

The most similar congener to *Patania clava* sp. n. is *Patania iopasalis* (Walker, 1859). However, the wingspan of *Patania iopasalis* is smaller with 21.0–30.0 mm (Fig. 5); the labial palpus has no brown patch near the base but a big brown patch near the apex (Fig. 6), the male antenna has ventral cilia about one-fourth the length of the diameter of flagellomere; in the male genitalia the short broad sheet-like gnathos lacks setae on the apex (Fig. 7), the phallus has an ovate sclerotized structure protruding from apex, and a long thick needle-like cornutus is embedded in a cluster of spicular cornuti (Fig. 8). *Patania clava* sp. n. is also similar to *Patania obfuscalis* (Yamanaka, 1998) in appearance. The latter can, however, be distinguished by its serrated postmedial line of forewing; in the male genitalia by the middle costa slightly inflated and bearing a cluster of setae, the vestigial gnathos, the board-like sclerotized, apically tapering structure protruding from apex, and a short broad thorn-like cornutus near a cluster of spicular cornuti.

**Figures 9–10.**
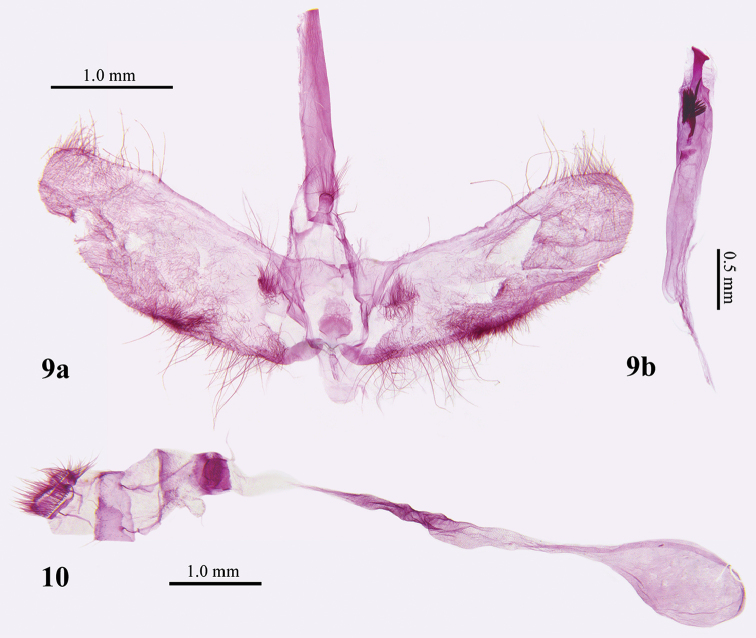
Genitalia of *Patania clava* sp. n. **9** Male, Holotype **a** Without phallus **b** Phallus, Holotype **10** Female, paratype.

The new species is only known from Diaoluo Mountain of Hainan Island at present. *Patania iopasalis* is widely distributed in south Asia, southeast Asia, east Asia, including Hainan Island, China, the Caroline Islands, New Guinea, Australia and Guatemala (Hampson 1896, 1898; Klima 1939; Xu 2015). *Patania obfuscalis* is distributed in Nepal and some areas of China, excluding Hainan Island.

## Supplementary Material

XML Treatment for Patania clava
